# A Rare Association: Autoimmune Hemolytic Anemia With Indolent T-Cell Prolymphocytic Leukemia

**DOI:** 10.7759/cureus.7994

**Published:** 2020-05-06

**Authors:** Hafiz A Yahya, Nadia Khan, Stefan Barta, Reza Nejati

**Affiliations:** 1 Pathology, Fox Chase Cancer Center, Philadelphia, USA; 2 Hematology/Oncology, Fox Chase Cancer Center, Temple University Health System, Philadelphia, USA; 3 Hematology/Oncology, Abramson Cancer Center, University of Pennsylvania, Philadelphia, USA; 4 Pathology/Hematopathology, Fox Chase Cancer Center, Philadelphia, USA

**Keywords:** autoimmune hemolytic anemia, indolent, t-cell prolymphocytic leukemia, rare

## Abstract

The association of warm autoimmune hemolytic anemia (wAIHA) with various lymphoproliferative disorders is well reported in the literature. But the association of wAIHA with T-cell prolymphocytic leukemia (T-PLL), a very rare lymphoproliferative disorder, has never been reported. A 71-year-old man was in his usual state of health until three years ago when he developed intermittent bouts of worsening anemia associated with mild peripheral blood lymphocytosis. He was diagnosed with wAIHA and steroid therapy was initiated, resulting in an improvement in the hemoglobin level of the patient. His lymphocyte count remained persistently elevated but he did not develop any malignancy-related signs or symptoms. A diagnosis of ‘indolent’ T-cell prolymphocytic leukemia (small cell variant) was made by combining distinctive clinical, morphologic, immunophenotypic, and cytogenetic analysis. His wAIHA went into complete remission and steroid therapy was successfully tapered off. He has not required any treatment for his T-PLL during the last two years' follow-up.

## Introduction

T-cell prolymphocytic leukemia (T-PLL) is characterized by the proliferation of mature, small to medium-sized prolymphocytes. It is a rare lymphoproliferative disease occurring more frequently in old males. It is an aggressive disease that commonly presents with splenomegaly, lymphadenopathy, hepatomegaly, and peripheral blood lymphocytosis. It can present as an ‘indolent’ disease as well [1-7]. Autoimmune hemolytic anemia (AIHA) is characterized by the production of autoantibodies against blood cell antigens destroying red blood cells. Its most common subtype is warm autoimmune hemolytic anemia (wAIHA). Lymphoproliferative disorders are one of the most common diseases associated with wAIHA. The most common lymphoproliferative disorders associated with wAIHA are chronic lymphocytic leukemia (CLL), non-Hodgkin's lymphoma (NHL), and classic Hodgkin's lymphoma (cHL) [8-14].

To the best of our knowledge, the association of wAIHA with T-PLL has never been reported in the literature. We describe here a rare association of wAIHA with indolent T-PLL.

## Case presentation

**Figure 1:**
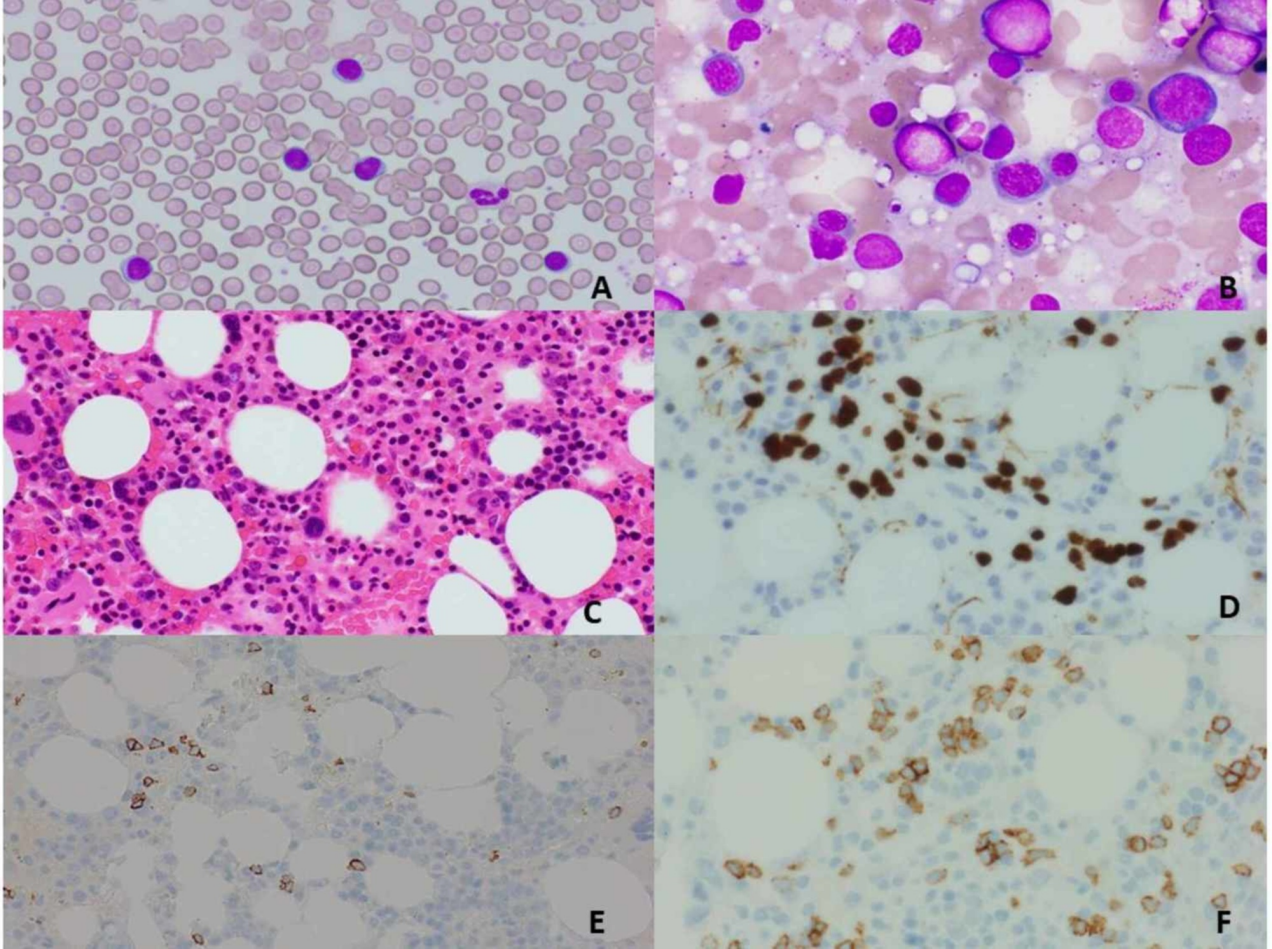
Peripheral blood smear (A) and bone marrow aspirate (B) showing small lymphocytes with round nuclei. Bone marrow H&E stain (C) revealing hypercellular marrow. bone marrow immunohistochemistry revealing that lymphocytes are positive for TCL1 (D), CD20 (E), and CD3 (F) H&E: hematoxylin and eosin; TCL: T-cell leukemia; CD: cluster of differentiation

**Figure 2:**
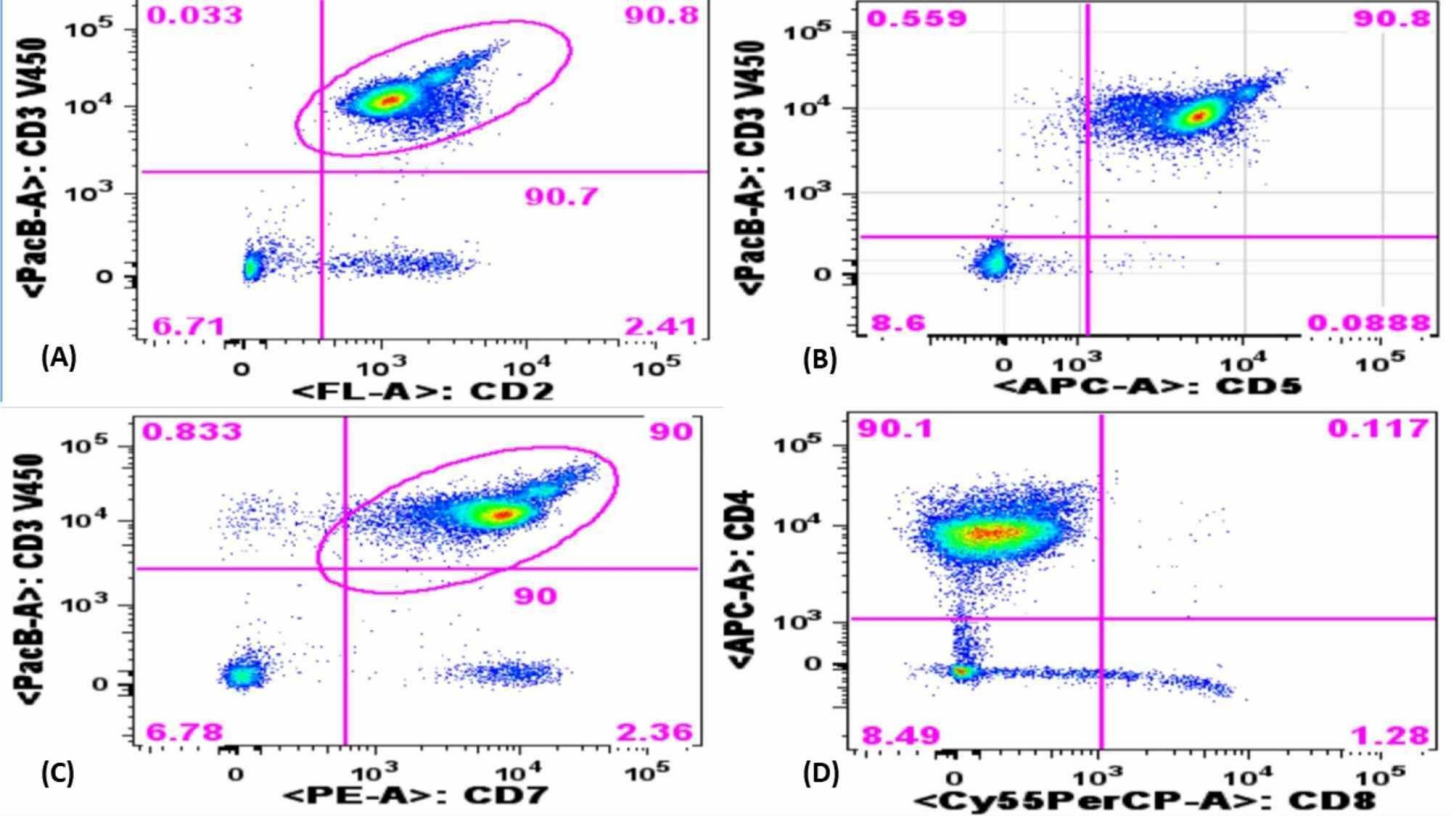
Peripheral blood flow cytometry demonstrating atypical T-cell population positive for (A) CD2, (B) CD5, (C) CD7, and (D) CD4 CD: cluster of differentiation

**Figure 3:**
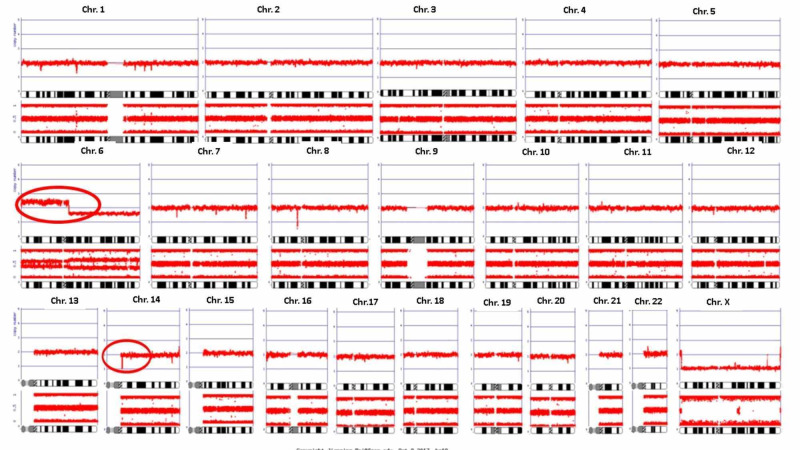
Chromosomal microarray analysis revealing loss of chromosome segment 6q, gain of chromosome segment 6p in mosaic state, and heterozygous deletion of T-cell receptor alpha variable region on chromosome 14

**Figure 4:**
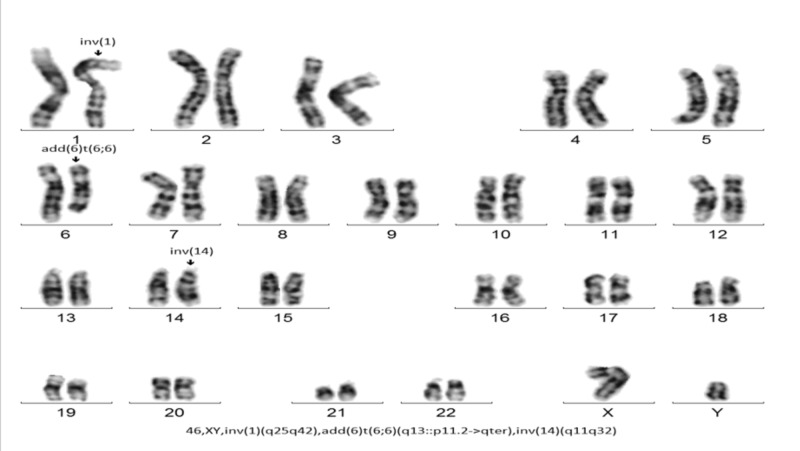
Patient's karyotype

**Figure 5:**
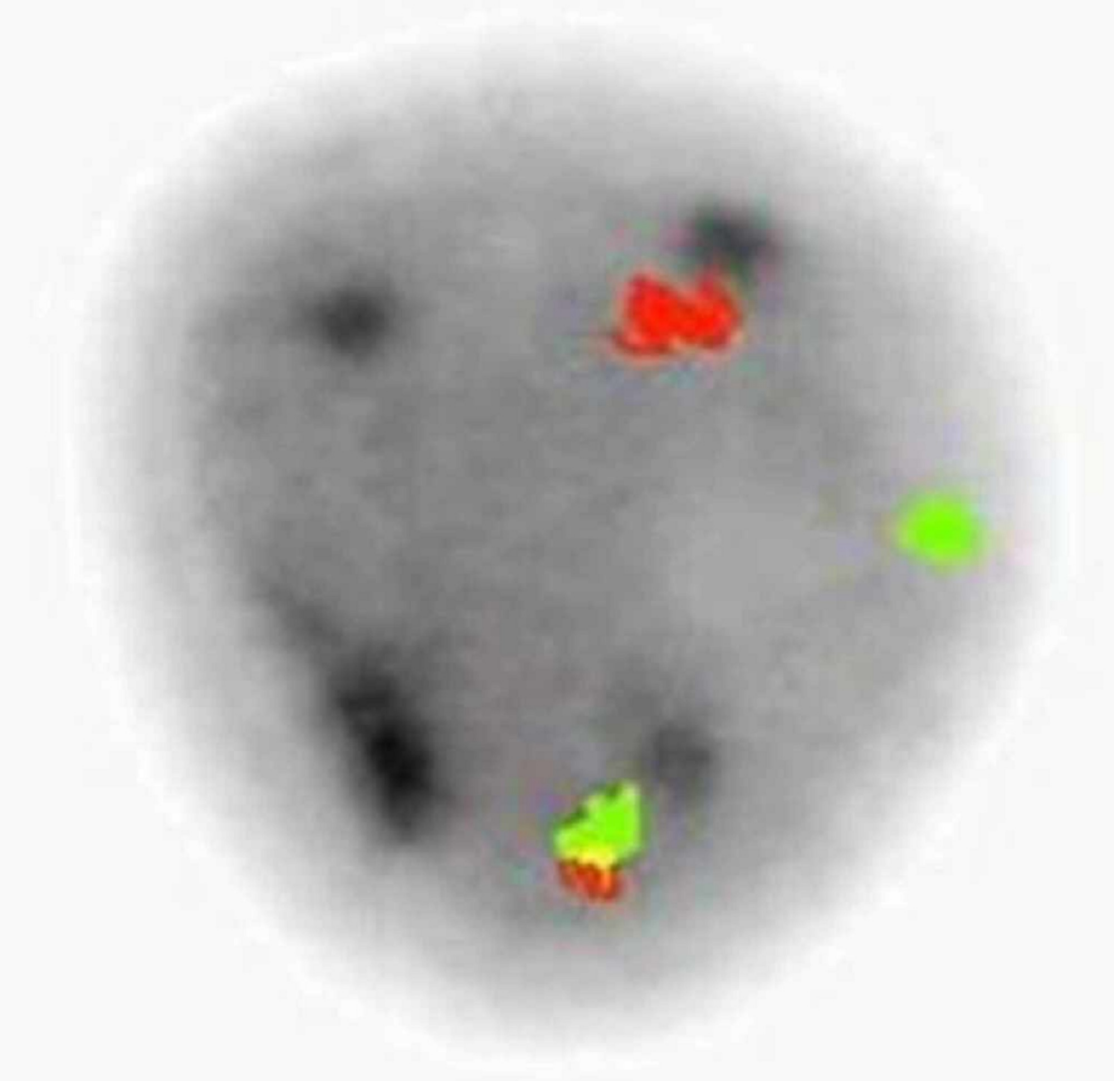
TCR-alpha/delta FISH study FISH study using dual-colored TCR alpha/delta (14q11.2) break-apart probe, with the 5' region labeled in red and the 3' region in green. In a normal chromosome 14, the 5' and 3' regions are contiguous, resulting in a single yellow or overlapping red/green signals; the inv(14) or TCR alpha/delta gene rearrangement splits the locus, resulting in separate red and green signals. FISH: fluorescence in situ hybridization; TCR: T-cell receptor

A 71-year-old man returned for a follow-up evaluation for the management of his autoimmune hemolytic anemia and underlying T-cell prolymphocytic leukemia. He was asymptomatic for the last two years. His past medical history included essential hypertension, obesity, hyperlipidemia, diabetes, and gout. Three years ago, he developed fatigue and his blood work revealed a hemoglobin of 6.6 g/dl along with a mildly elevated lymphocyte count. He underwent colonoscopy and upper gastrointestinal (GI) endoscopy, which was unremarkable. Two years ago, while taking an antibiotic (Sulfa drug) for a presumed upper respiratory tract infection, his hemoglobin dropped again to 6.5 g/dl and did not improve with blood transfusion. At that time, the direct antiglobulin test (DAT) was positive and revealed anti-Kell antibodies on elution. Cold agglutinins were not detected. Serum protein electrophoresis was normal. antinuclear and antiphospholipid antibodies were not present. He was diagnosed with warm AIHA (wAIHA) and was started on prednisone (1 mg/kg). His hemoglobin level improved but lymphocytosis persisted. CT scan revealed no lymphadenopathy or hepatosplenomegaly. Peripheral blood smear revealed lymphocytosis with mostly small lymphocytes with round nuclei and small nucleoli. Bone marrow biopsy revealed a hypercellular marrow (95%) with infiltration by predominantly small lymphocytes. Immunohistochemical stains on bone marrow core biopsy revealed the lymphocytic infiltrate to be composed primarily of cluster of differentiation 3+ (CD3+)/TCL1+ T-cells (10%-15% of the nucleated cells) (Figure 1). Flow cytometry demonstrated an abnormal CD4/CD8 ratio of approximately 70:1 without aberrant immune-phenotype. T-cells were found to be CD2+, CD5+, and CD7+ (Figure 2). A T-cell receptor gene rearrangement study of the peripheral blood was positive for T-cell receptor (TCR) beta and gamma gene rearrangement. Chromosomal microarray analysis revealed a loss of chromosome segment 6q, a gain of 6p in a mosaic state, and heterozygous deletion of the T-cell receptor alpha variable region on chromosome 14 (Figure 3). Conventional cytogenetics showed an abnormal mosaic male karyotype: 46,XY,inv(1)(q25q42),add(6)t(6;6)(q13::p11.2->pter),inv(14) (q11q32)[cp17]/46,XY*[5] *(Figure 4). Fluorescence In situ hybridization (FISH) identified the T-cell receptor alpha/delta gene rearrangement at 14q11.2 (Figure 5).

Differential diagnosis

Antibiotic-associated anemia was ruled out due to persistently elevated anti-Kell antibodies after the discontinuation of the drug.

Treatment

Steroid therapy resulted in the complete remission of wAIHA and the patient was successfully tapered off. He never got any treatment for his T-PLL.

Outcome and follow-up

The patient continues to remain asymptomatic with relatively stable lymphocyte count. His most recent laboratory results show hemoglobin=15.5 g/dl (reference range=14.0-17.0), red cell count=5.42 per pl (reference range=4.20-5.70), white cell count=15.7 per mm^3^ (reference range=4.5-11), mean corpuscular volume=86.6 fl (reference range=80.2-99.4), platelet count=266,000 per 𝜇l (reference range=150,000-450,000), hematocrit=47.0% (reference range=39.0-50.0), mean corpuscular hemoglobin concentration= 33.0 pg/cell (reference range=33-36), and red cell distribution width=14.2% (reference range=11.5-14.5).

## Discussion

T-cell prolymphocytic leukemia (T-PLL) is aggressive T-cell leukemia characterized by the proliferation of mature small to medium-sized prolymphocytes. It is a rare disease accounting for 2% of cases of mature lymphocytic leukemias in adults aged >30 years. Its median age of presentation is 65 years (range=30-94 years). It presents more frequently in adult males, with splenomegaly, lymphadenopathy, hepatomegaly, and, occasionally, with dermal infiltration and serous effusion [1-2]. The indolent phase of T-PLL has also been described in which the patient remains clinically silent with the background of peripheral blood lymphocytosis. The average period for this phase is 33 months (6-103); after that, unfortunately, it transforms into a rapidly fatal aggressive disease [3-5]. However, there is a case report that the patient remained in the indolent phase for 18 years, which is strikingly exceptional [6]. Here, we have described a patient who developed indolent T-PLL associated with AIHA.

We seek to add indolent T-PLL into the list of diseases associated with AIHA. We conducted a comprehensive search by PubMed of all the literature published on this entity so far. We found multiple reported cases of indolent T-PLL, which is a very rare disease, however, we did not find any case revealing its association with AIHA.

There are some important characteristics of our case that suggest that indolent T-PLL can be involved in the pathogenesis of wAIHA: (1) Peripheral blood lymphocytosis was present with anemia when the patient presented for the first time three years ago. Moving ahead in time, the patient developed intermittent bouts of anemia with the background of consistently elevated lymphocyte count. Based on this finding, one can suggest that T-PLL has temporally preceded the development of wAIHA. (2) It is well-reported in the literature that 50% of cases of AIHA are due to the presence of a secondary underlying cause [15]. However, our extensive workup did not reveal evidence of any secondary cause except T-PLL.

Finally, our patient’s AIHA went into complete remission after using steroids for eight months. This suggests that AIHA associated with T-PLL can respond to steroids, contrary to AIHA associated with other malignancies [16]. Here, it is worth mentioning that we have described just one case of AIHA associated with T-PLL that has shown such a response to steroids. Whether it is true for all such cases needs the addition of similar cases in the literature. Moreover, some malignancy-related AIHA disappears when underlying malignancy is treated [15]. Whether this is true for T-PLL-associated AIHA remains to be elucidated.

## Conclusions

T-cell prolymphocytic leukemia (T-PLL) should be added to the list of underlying diseases associated with wAIHA. In addition, steroid use caused the complete remission of T-PLL-associated wAIHA in our patient. To conclude, our patient’s indolent T-PLL required no treatment from the time the disease was recognized.

## References

[1] WHO WHO (2001). Pathology and Genetics of Tumours of Haematopoietic and Lymphoid Tissues. Iarc.

[2] Matutes E, Brito-Babapulle V, Swansbury J (1991). Clinical and laboratory features of 78 cases of T-prolymphocytic leukemia. Blood.

[3] Tirado CA, Starshak P, Delgado P, Rao N (2012). T-cell prolymphocytic leukemia (T-PLL), a heterogeneous disease exemplified by two cases and the important role of cytogenetics: a multidisciplinary approach. Exp Hematol Oncol.

[4] Garand R, Goasguen J, Brizard A (1998). Indolent course as a relatively frequent presentation in T‐prolymphocytic leukaemia. Br J Haematol.

[5] Clément Janot JF, Borie C, Lefevre E, Bennaceur‐Griscelli A, Turhan AG, Aumont C (2019). Clinically silent indolent T‐cell leukemia. Clin Case Rep.

[6] Adediran S, Cornfield D, Bagg A, Agostino N (2016). An extremely indolent T‐cell leukaemia: an 18‐year follow‐up. J Community Support Oncol.

[7] Dearden C (2012). How I treat prolymphocytic leukemia. Blood.

[8] Michel M (2011). Classification and therapeutic approaches in autoimmune hemolytic anemia: an update. Expert Rev Hematol.

[9] Lechner K, Jäger U (2010). How I treat autoimmune hemolytic anemias in adults. Blood.

[10] Mauro FR, Foa R, Cerretti R, Giannarelli D, Coluzzi S, Mandelli F, Girelli G (2000). Autoimmune hemolytic anemia in chronic lymphocytic leukemia: clinical, therapeutic, and prognostic features. Blood.

[11] Valent P, Lechner K (2008). Diagnosis and treatment of autoimmune haemolytic anaemias in adults: a clinical review [Article in German]. Wien Klin Wochenschr.

[12] Agrawal K, Alfonso F (2017). A rare association of autoimmune hemolytic anemia with gastric adenocarcinoma. Case Rep Oncol Med.

[13] Michel M (2008). Characteristics of warm autoimmune hemolytic anemia and Evans syndrome in adults [Article in French]. Presse Med.

[14] Zanella A, Barcellini W (2014). Treatment of autoimmune hemolytic anemias. Haematologica.

[15] Salmeron G, Molina TJ, Fieschi C, Zagdanski AM, Brice P, Sibon D (2013). Autoimmune hemolytic anemia and nodular lymphocyte-predominant Hodgkin lymphoma: a rare association. Case Rep Hematol.

[16] Puthenparambil J, Lechner K, Kornek G (2010). Autoimmune hemolytic anemia as a paraneoplastic phenomenon in solid tumors: a critical analysis of 52 cases reported in the literature [Article in German]. Wien Klin Wochensch.

